# Supplementary material for: Are carbon emissions trading and green financial instruments synergistic? -Comprehensive quantitative research based on content analysis
Source: PLoS One. 2024 Mar 7;19(3):e0298601. doi: 10.1371/journal.pone.0298601 (PMC10919601; doi:10.1371/journal.pone.0298601)
Supplement: S1 Data — (ZIP) [file pone.0298601.s001.zip › Data/地级市Z_F工作报告.zip!/║╙─╧/╔╠╟≡╩╨/└┤╫╘╣1⁄2╓┌║┼í╛┬φ┐╦╩2╛▌═°í┐_2008.docx]

各位代表：

　　现在，我代表市人民政府，向大会作政府工作报告，请予审议，并请各位政协委员和其他列席会议的同志提出意见。

一、去年工作回顾

　　2007年，在省委、省政府和市委的正确领导下，在市人大、市政协的监督支持下，市政府和全市人民一道坚持以邓小平理论和“三个代表”重要思想为指导，认真学习贯彻党的十七大精神，全面落实科学发展观，扎实工作，开拓进取，圆满完成了市三届人大一次会议确定的各项目标任务。全年实现生产总值783．05亿元，增长13．3％；财政一般预算收入26．7亿元，增长28．1％；全社会固定资产投资400．7亿元，增长33．2％；全年社会消费品零售总额234．1亿元，增长17．6％；城镇居民人均可支配收入10160元，增长21．8％，农民人均纯收入3248元，增长18．3％；金融机构年末各项存款余额502．8亿元，增长10．8％，贷款余额411．3亿元，增长16．6％。

　　工业化进程明显加快。顺应国家宏观调控，不断优化经济结构，坚定不移地走新型工业化道路。全部工业实现增加值275．9亿元，增长18．6％，其中规模以上工业实现增加值183．9亿元，增长23．2％，工业对经济增长的贡献率提高到45．8％。重点骨干企业规模和效益同步提高，规模以上工业企业发展到450家，销售收入超亿元企业达到82家，超10亿元企业7家。永煤集团实现了在全国同行业效益、人均利税、人均收入三项第一，神火集团跨入中国企业500强，商电铝业集团迈入全国同行业10强。产业链条进一步拉长，煤化工、铝深加工等产业链得到重点培育，永煤集团新桥矿、神火集团4万吨工业型材铸锭、龙宇煤化工年产50万吨甲醇等工业项目相继建成投产，成为新的经济增长点。品牌战略取得明显成效，20多个产品和商标获得国家、省“名牌产品”、“优质产品”和河南省“著名商标”称号。工业集聚区建设取得重大进展，全市现有11个工业集聚区，总规划面积190．9平方公里，建设标准厂房53．7万平方米，入驻固定资产投资1000万元以上工业项目153个。重点项目建设成效显著，全年安排重点建设项目187个，完成投资131．3亿元，其中86个重点项目竣工投产，新增原煤开采能力120万吨，纺纱能力23万锭，家用电器生产能力40万台，服装加工能力270万套，商丘经济发展的后劲大大增强。

　　“三农”工作再上新台阶。从加强农业基础入手，把促进粮食增产和农民增收作为首要任务，用活用足政策，不断加大投入，取得了显著成效。农业基础地位不断加强，粮食生产再创历史新高，全年粮食总产115亿斤，居全省第二位，粮食总产连续两年突破百亿斤大关，被评为全国粮食生产先进市。农业产业化经营取得明显成效，以“五类十大基地”为载体，大力发展农畜产品精深加工，市级以上农业产业化重点龙头企业达到185家，其中省级24家，居全省第三位。76个农产品生产基地通过省级无公害农产品基地认定，居全省第一位。规模养殖小区达到258个，规模养殖场达到3646个。大力发展劳务经济，积极开展农民培训，实现劳务输出200万人次，创劳务收入120亿元。农村基础设施建设得到加强，新修“村村通”道路2725公里，所有行政村通油路或水泥路。在全省率先完成“户户通电”工程，消除了“盲点村”和“无电户”。完成“空心村”整治123个。新建农村安全饮水工程36处，解决了21．24万人安全饮水问题。新建沼气池5万座。农机总动力达到1040万千瓦。农田水利基本建设连续七年荣获省“红旗渠精神杯”。新农村建设扎实推进，组织实施了“百村示范、千村带动、全面推进”工程，开展“为社会主义新农村建设办实事”活动，实施了“小拆迁、大整治”试点工作，村容村貌得到有效改善。支农惠农政策得到全面落实，共发放补贴资金5．15亿元，发放“两免一补”资金3．7亿元，受惠学生达154万人，极大地调动了广大农民的生产积极性。

　　第三产业发展提速。充分利用商丘独特的交通区位优势和产业基础，瞄准市场，围绕扩大规模、提升层次、增强实力，不断发展壮大第三产业。全市第三产业增速18％，高于全省4．5个百分点。现代物流体系加速完善，新建钢材大市场二期、中意建材市场二期等物流项目21个。全市注册登记物流企业72家，专业批发市场145个，各类农产品批发市场675个，其中年交易额超亿元批发市场14个。商丘农产品中心批发市场年交易额超百亿元，进入全国农产品批发市场前三强。证券、保险、信息产业发展迅速，实现股票交易额495亿元，各类保险公司发展到18家。各类移动通讯用户达到254．5万户，固定电话用户120．5万户，互联网宽带用户9．5万户，全市80％以上的行政村通了宽带。旅游产业高速发展，隋唐大运河商丘段和商丘古城申遗工作有序展开，永城芒砀山汉文化景区综合整治、商丘古城保护与开发迈出坚实步伐。全年共接待游客502万人次，旅游收入9．27亿元，分别增长42．6％和22．3％，均创历史最高水平。

　　文化名市建设迈出坚实步伐。坚持以体制改革创新为动力，以文化事业与产业发展并重为目标，大力挖掘和弘扬“商”文化，荣获“华商之都”城市品牌。商丘演艺集团在全国产生广泛影响，王公庄画虎村被命名为国家和省文化（美术）产业示范基地，“木兰传说”入围国家级非物质文化遗产名录。建设农村文化大院121个、乡镇（社区）文化站17个。群众性体育活动蓬勃开展，我市被确定为2008奥运会火炬传递重点城市，成功举办了市第三届运动会，顺利承办了全国铁人三项锦标赛。文化体制改革取得新突破，组建了商丘日报报业集团，对商丘影剧院等单位进行了改制，市图书大厦、图书馆、体育馆等建成并投入使用，商丘广电中心开工建设，为全市文化建设的繁荣与发展打下了良好基础。

　　城镇化水平得到明显提高。统筹中心城市、县城、小城镇协调发展，高起点编制完成了《商丘城市发展战略规划》和城市交通、住房建设、消防、园林绿化等100多项专项规划，各县（市）总体规划修编全部完成，乡镇总体规划修编完成率达到90％。实施了34条市政道路新建和改造工程。市区达到了日处理垃圾能力720吨，供热能力800万平方米，天然气用户1．7万户。县县建成污水处理厂、垃圾处理场。房地产业健康发展，全市房地产开发投资26．6亿元，增长38．8％。全市城镇建成区面积344．6平方公里，城镇人口259．8万，城镇化率达到31．5％。实施城市精细化管理，在全省率先开展了违法用地、违法建设集中整治活动，卫生、交通、经营“三大秩序”整治取得明显成效，人居环境显著改善。生态环境建设成效突出，5个国控断面水质达标率为95．8％，饮用水源地水质达标率为100％，市区空气质量优良天数为325天，优良率89％。节能减排取得实效，万元GDP能耗降低到1．412吨。盘活城镇存量土地9300亩，连续8年实现耕地占补平衡。2007年，顺利入选“中国特色魅力城市200强”和“中部最佳投资城市”。

　　招商引资工作取得重大突破。“招商引资提高年”活动扎实推动，主动组团参加中博会、广交会、深圳高交会等国内大型经贸洽谈活动，瞄准“长三角”、“珠三角”、“环渤海湾”区域强力招商，全年引进招商引资项目1464个，其中新建项目931个，到位资金118．3亿元；续建、改扩建项目533个，到位资金106．2亿元。直接利用外来投资142．6亿元，其中引进省外资金105亿元，被评为全省利用省外资金先进市。通过招商引资，新建投资千万元以上项目673个，引进规模以上工业项目309个，引进外向型项目62个，为商丘的发展备足了后劲。

　　各项改革持续深化。工业企业改革以深化国有资产管理体制改革、扶持优势产业和大型骨干企业做大做强为重点，对振华玻璃厂、冰熊冷柜、商桐公司等企业实施了重组和资产转让，促进了企业经营机制的转换，增强了企业活力。推进企业依法破产，申报政策性破产项目3个，破产金额6亿元。城市建设投融资体制改革大力推进，中心城区热力、燃气、供水、地下信息管网等市政基础设施建设分别与加拿大凯迪实业、新奥集团、香港远耀、北京信通实行了合作。市污水处理厂与重庆康达环保公司实施了资产和经营权转让。市公交总公司完成了改制，市内35条公交线路全部实现市场化运作。财政金融体制改革逐步深入，财政预算管理制度、国库集中支付制度和“乡财县管”体制改革不断完善，农村信用社改革取得阶段性成效，城信社向商业银行改制工作进展顺利。打造“诚信商丘”，深入开展“重合同、守信用”、“创建全国金融生态环境模范试点城市”活动，各类信用担保体系不断完善。

　　和谐社会建设全面推进。教育事业快速发展，“两基”工作顺利通过国家级验收，商丘职业技术学院被确定为国家示范性高等职业院校。各县（市、区）全部建立新型农村合作医疗制度，农民参合率达到82．2％。全市所有乡镇卫生院基本改造一遍，标准化社区卫生服务中心（站）达到45个。社会保障体系进一步完善，城镇新增就业9．5万人，下岗失业人员实现再就业3．6万人。提高了城乡低保标准，发放低保资金1．7亿元，32．5万人得到最低生活救助。拨付城乡医疗救助资金1918万元，缓解了特困家庭看病难问题。新建、扩建敬老院100所，五保户集中供养率达到30％。全市60岁以上老人全部享受免费乘坐公交车。改善低收入群体住房条件，新建经济适用住房19．7万平方米。开展产品质量和食品药品安全专项整治活动，保障了人民群众消费安全。人口和计划生育管理服务水平不断提高，人口自然增长率控制在4．6‰以内。加强社会治安综合治理，妥善处理涉及群众利益的信访突出问题，严格落实安全生产责任制，社会治安和安全生产形势平稳。军政军民团结不断加强，第五次荣获“全国双拥模范城”称号。

　　依法行政迈出新的步伐。坚持科学民主决策，推进依法行政，加强行政监督，不断提高政府工作效能。自觉接受人大的工作监督、法律监督和政协的民主监督，办理人大代表建议236件，政协委员提案498件。深化行政审批制度改革，取消行政许可、非行政许可项目420项。公务员教育培训和管理法制化建设进一步加强。坚持不懈地开展反腐败斗争和加强政风建设，治理商业贿赂工作取得良好成效，依法查处违法违规案件和失职渎职行为，惩处腐败分子。社会管理和公共服务职能得到强化，人事、编制、审计、科技、知识产权保护、统计、粮食、供销、检验检疫、物价、海关、盐业、烟草、邮政、妇女儿童保护、残疾人康复、外事侨务、机关事务、民族宗教、地方史志、档案、人防、气象、防震减灾等工作都取得了新的成绩。

　　各位代表，这些成绩的取得，令人鼓舞而又来之不易。这是在改革开放以来、特别是商丘建市十年来历届班子持续奋斗的结果，是全市人民万众一心、励精图治、奋发进取、共同努力的结果，是省委、省政府和市委正确领导的结果，是人大、政协和社会各界监督支持的结果，标志着我市发展已经踏上了新的起点，跨上了新的征程。在此，我代表市人民政府，向全市广大工人、农民、知识分子和干部，向解放军、武警、消防官兵、政法干警，向各民主党派、人民团体和离退休老同志致以崇高的敬意！向所有关心、支持商丘发展的社会各界人士及海内外朋友，向在我市创业发展的投资者、合作者、建设者表示衷心的感谢！

　　在肯定成绩的同时，我们也清醒地认识到，经济社会发展中仍存在一些问题和不足。主要表现在：经济总量小、人均水平低，结构性矛盾仍较突出；经济外向度偏低，节能减排压力加大，资源环境约束问题日益突出；县域经济综合实力不强，城乡居民收入水平不高，社会事业发展相对滞后，改善民生的任务艰巨；新时期社会稳定的任务十分繁重；政府自身建设需要进一步加强等。对此，我们一定要高度重视并采取切实有效措施，认真加以解决。

二、当前的形势和工作要求

　　各位代表，2008年是全面贯彻落实党的十七大精神的第一年，也是改革开放30周年和举办北京奥运会的喜庆之年。新的一年，我们面临许多难得机遇。就国际而言，经济全球化深入发展，国际市场更为开放，科技创新成果和资金、人才等生产要素的流动更加广泛迅速，国际产业转移进一步加速，为我们加快发展提供了新的空间；就国内而言，我国将进一步扩大国内消费需求，更加重视“三农”工作，加快推动中部崛起战略实施，推动区域协调发展；就省内而言，省委、省政府加快黄淮四市发展的战略部署，为我们加快发展创造了条件。因此，做好今年的各项工作有利条件很多，意义十分重大。我们要按照“思想大解放、经济大开放、全民大创业”的总要求，坚定信心、振奋精神、奋发有为、加快发展，促进商丘经济社会发展再上新台阶。

　　做好政府工作，把商丘的各项事业办好，在总体要求上必须继续牢牢把握以下几点：

　　（一）始终把解放思想作为总开关。思想是行动的先导，改革开放的历程表明，没有思想的大解放，就没有新一轮的经济大发展。在当前经济体制全面转轨、经济发展方式加快转型、产业和资本加速转移的背景下，我们的发展理念应更加开放，视野应更加开阔，努力站得更高一些，看得更远一些，想得更深一些，推动经济社会在更新要求上的又好又快发展。要着力冲破一切不合时宜的观念束缚，着力革除一切与形势发展不相适应的思维定式，坚持从实际出发，真正把解放思想体现在工作上，落实到行动中，努力以新观念、新思路、新办法谋划发展方略，破解瓶颈制约，在应对挑战中拓展新路，在抢抓机遇中奋勇争先，在推动发展中勇辟捷径，形成干事创业的思想合力。

　　（二）始终把加快发展作为第一要务。发展是时代的主题，是执政兴国的第一要务，是破解商丘一切难题的金钥匙。只有树立强烈的忧患意识、机遇意识和责任意识，聚精会神搞建设，一心一意谋发展，才能实现符合科学发展观要求的速度、质量和效益。在工作上，无论是谋划决策，还是推动落实，都要自觉地以科学发展观为统领，准确把握商丘市情，吃透发展的阶段性特征，着力研究解决影响科学发展的关键问题，只要是符合国家产业政策的发展，只要是符合节能、节地和环保要求的发展，只要是有质量、有效益的发展，能发展多快就发展多快，以实际行动推动科学发展观在我市的具体化贯彻落实。

　　（三）始终把项目建设作为主要抓手。在区域经济发展中，项目具有龙头地位、辐射带动作用，是引领经济社会发展的突破口。要增强项目意识，坚持项目先导，始终把项目建设作为抓发展、促跨越的根本举措，切实做到以项目求突破，以项目促发展，以项目论实绩，千方百计争取国家投资项目，全力以赴抓好招商引资项目，集中智慧激活民间创业项目，切实做到调动各方面力量运作项目，集中优势资源投入项目，优化软硬环境建设项目，用项目带动投资的扩张，用项目促进产业的升级，用项目加快实力的提升和经济社会的持续协调发展。

　　（四）始终把改善民生作为核心任务。在执政为民的新要求和发展社会主义市场经济的新形势下，解决民生问题就是最大的政治，改善民生问题就是最大的政绩。要坚持以人为本，多办利民之事，多行惠民之举，围绕人民群众的所思所盼，多办实事，多解难事，切实把政策和财力、物力、精力向公共服务领域倾斜，向社会事业倾斜，向社会保障体系倾斜，向维护社会公平正义、保障公共安全方面倾斜，着力解决涉及教育卫生、就业创业、社会保障、住房福利、环境保护和社会稳定等群众最关心、最直接、最现实的利益问题，让更多的民众共享改革发展成果，促进社会和谐与全面进步。

　　（五）始终把改革创新作为发展动力。改革开放是强国之路，锐意创新是发展之源。要坚持用开放的视野、改革的思路、创新的举措，研究新情况、解决新问题、开创新境界。要改革体制机制，形成有利于科学发展的制度保障，切实让政府的职责权力服务于全局发展的需要，让政府的行政行为服从于法律法规的要求，让政府的工作质量体现在改善民生的成效之中。要大力营造尊重知识、尊重人才、支持创造、鼓励创新的社会氛围，不断增强自主创新能力，提高经济社会发展的综合竞争力。

三、2008年的主要任务

　　今年政府工作的指导思想是：高举旗帜，科学发展，顺应调控，务实推进，努力在转变经济发展方式上有新进展，在坚持新型工业化道路上迈出新步伐，在统筹城乡协调发展上取得新成效，在着力改善民生上体现新作为，在继续扩大改革开放上实现新突破，促进经济社会又好又快发展。

　　今年全市经济社会发展的主要预期目标是：全市生产总值增长13％；全社会固定资产投资增长20％；地方财政一般预算收入增长15％；社会消费品零售总额增长15％；外贸进出口增长16％；合同利用外商投资增长25％；单位生产总值能耗降低5．4％；化学需氧量、二氧化硫排放量分别下降10．9％和1．6％；城镇居民人均可支配收入增长8％，农民人均纯收入增长7％。

　　今年重点做好以下九个方面的工作：

　　（一）以“工业立市”战略为主导，加快推进新型工业化进程。坚持把工业放在经济工作的突出位置，通过调整工业经济结构，做大做强做优工业，着力培育壮大经济增长点，激发经济发展活力，尽快形成以优势产业为主导、以重点企业为支撑、以工业集聚区为载体的发展新格局。

　　扶持壮大优势企业。坚持扶优扶强，支持一批核心企业向大而强的方向发展。力争全市实现销售收入超亿元工业企业达到100家以上，规模以上工业企业实现利税总额100亿元以上。按照有龙头企业、有骨干产品、有比较优势的原则，通过项目带动，大力培育有竞争力的企业。加快发展煤化工、农副产品深加工、商贸物流等特色产业和骨干企业。对具有市场优势和发展潜力的中小企业，加大扶持力度，加快发展步伐，尽快成为新的骨干企业。推进工业企业改革和战略重组，重点做好恒兴纸业与省投资集团、冰熊冷柜与浙江华美、冰熊冷藏车与海泰实业等企业重组合作，积极推进科迪集团、蓝牌酒业等企业的对外战略重组。加快推进永煤股份、张弓酒业、华星面粉、科迪集团等企业上市步伐。

　　加快建设工业集聚区。按照“布局集中、用地集约、产业集聚”的要求，利用好工业集聚区的优惠政策，突出发展优势产业和龙头企业，努力形成产业集聚。大力推进标准厂房建设，确保每个县新增标准厂房面积平均2万平方米以上。对工业集聚区建设实行目标管理，在科学制定发展规划，加快基础设施建设步伐的同时，进一步优化功能布局，大力开展产业招商，引导同类企业集聚，尽快形成项目的突破，提高产出效益。

　　大力发展民营经济。以全民参与、全民创业为主线，以明确的政策导向，引导上项目，鼓励办企业，培育更多的市场主体。加强民营企业人才培训、创业辅导、融资担保等服务体系建设，以服务促发展。组织实施中小企业诚信工程，积极开展民营企业银企合作，大力争取金融机构对民营企业的信贷支持。扶持引导在外完成积累、事业有成的务工人员返乡创业，带动地方经济发展。高度重视并切实加强民营企业家队伍建设，不断提高其产品开发能力、市场营销能力和企业管理能力，引导民营企业大胆推动企业体制、技术和管理创新，打造知名品牌，支持做大做强。

　　积极推进企业技术创新。突出企业技术创新主体地位，鼓励企业以市场为导向，开展针对性强的产学研合作，促进科技成果向现实生产力的转化。注重高技术、复合型人才的培养与引进，以重点产业、科研基地、科研项目为依托，培养一批科技骨干和创新型技术人才。继续实施名牌战略，重点扶持带动能力强、拥有自主知识产权的企业，扩大名牌产品生产规模和市场知名度，不断增强优势产业和产品竞争实力。

　　（二）强化农业基础地位，促进农业发展和农民增收。切实把“三农”工作摆在重中之重，以增加农民收入为核心，全面落实强农惠农政策，大力发展现代农业，着力推进农业结构调整，提高农业综合效益，加快社会主义新农村建设步伐。

　　稳定提高粮食综合生产能力。围绕打造全省粮食生产核心区，用足用活国家和省粮食生产各项扶持政策，保护和提高农民种粮积极性。抓好大型商品粮基地项目建设，实施农业综合开发，提高机械化耕作水平，搞好中低产田改造，使全年粮食播种面积稳定在1350万亩以上，粮食总产量稳定在500万吨以上，力争保持550万吨。

　　加快农业产业化发展。围绕龙头企业、基地建设、产品开发、市场营销等关键环节，深入推进农业产业化。重点抓好185家市级以上农业产业化重点龙头企业，增强辐射带动能力。大力推进农业标准化生产，实行农产品市场准入制度，提高农产品质量安全水平。调整和优化农业结构，推进规模化生产，突出发展规模种植和规模养殖，新增规模种植小区20个以上，规模养殖小区85个以上，不断提高规模效益。以科迪集团为龙头，加快推进奶业示范县项目建设，扶持乳制品加工业做大做强。积极发展农民专业合作社和农村服务组织，鼓励有实力的龙头企业、农村能人牵头兴办各类农村经济合作组织，畅通农产品流通渠道，逐步建立健全适应现代农业发展要求的市场流通网络。

　　推进农业科技进步。实施种子、植保、科技入户、科技丰产工程，加快农业科技成果转化。扩大测土配方施肥规模，抓好沃土工程综合示范基地建设。健全农业科技推广服务体系，鼓励农业科技人员通过有偿服务、技术入股、承包经营等形式，加快农业技术的推广和应用。积极探索基层动物防疫工作新机制，做好重大动物疫病防控工作。加强对农民机械操作、科学施肥、农作物病虫害防治等先进适用技术培训，提高农民科技素质。发挥职业院校优势，加强农村职业教育和技能培训，提高农民转移就业能力。

　　强化农业基础建设。加快优质粮食产业工程和标准化良田建设，加大土地开发整理复垦力度。继续搞好农田水利基本建设，实施林七、吴屯等水库大坝除险加固、“井井通电”和沼气建设工程，加强县乡公路建设和改造，进一步改善农业生产和农民生活条件。完成116个村的扶贫开发任务。

　　搞好村容村貌整治。根据村庄自然条件和经济发展状况，加快推进村庄建设和整治规划编制工作，分步组织实施，优化农村人居环境，改变农村整体面貌。对经济条件较好的村，重点提高建设和管理水平，发挥辐射带动作用。对基础薄弱、经济贫困的村，实施整村推进。对处于中间层次的村，加快推进饮水、道路、电力、文化、通讯等基础设施建设。

　　（三）以项目建设为突破口，努力提高对外开放水平。坚持把招商引资作为“天字号”工程　、“一把手”工程，持续展开招商攻势，狠抓重点项目落实，靠扩大开放增加有效投入，增创竞争优势，增强发展活力。

　　强力推进招商引资工作。坚持把特大项目、工业项目、高附加值项目、产业链项目作为招商引资的重点，特别注重引进战略投资者，实现经济发展的倍增效应。从严控制高投入、高能耗、高污染项目和企业的引进。按照蹲点招商、以商招商、落地招商、企业招商等多种形式，扩大招商队伍，改进招商方式，抓好境外招商。要切实组织好节会招商。今年要积极参加国家级和省级经贸洽谈活动，认真办好第二届中国?商丘华商国际文化节，做好2008年国际铁人三项洲际杯赛暨全国铁人三项冠军杯系列赛工作。要加强对招商项目巡回检查和监督活动，把签约项目的履约率、资金到位率、开工率、竣工投产率作为督查工作的重点，确保项目和资金落实到位。简化行政审批程序，完善重点招商项目领导负责制和联系人制度，切实维护投资者的合法权益，确保项目和资金引得来、留得住。

　　全力突破重点工程项目。坚持以项目促投资，以投资促发展，以发展促跨越，不断增强发展后劲。今年全市初步安排重点建设项目187个，总投资867．2亿元，计划投资165亿元。千方百计加快重点项目建设进度，对民权电厂一期、龙宇煤化工50万吨甲醇等91个项目，确保按计划建成投产。对神火集团薛湖矿等在建项目，排出阶段性进度和年度计划，加快建设进度。对龙宇煤化工年产20万吨醋酸等81个计划新开工项目，加大协调指导力度，实行全过程跟踪服务和管理，确保如期开工。对民权电厂二期、龙宇煤化工甲醇二期等项目，强力推进前期工作，积极创造条件，争取尽早开工建设。加快柘城煤田普查工作进度。增强项目意识，树立抓项目就是抓发展，服务项目建设就是服务经济发展的理念，把重点项目建设列入重要日程，增强抓项目的责任感和紧迫感，深入一线，深入基层，狠抓重点项目的落实。大力营造项目建设的浓厚氛围，实行重大项目建设领导联系制、专门班子负责制、进展情况定期通报制，纳入全市目标管理体系认真考核。落实奖励办法，大力宣传支持、服务项目建设的典型，依法严厉打击干扰、破坏重点项目建设的违法行为，公开查处损害项目建设环境的典型案件，为项目建设创造良好环境。

　　不断扩大经济外向度。以扩大自营出口为重点，加大对进出口企业产品补贴、企业融资等扶持力度，培育出口骨干企业和企业集团，优化出口商品结构，提高出口商品的档次和附加值，力争完成进出口总额3．6亿美元。加快国际劳务输出步伐，发展对外工程承包与劳务合作。充分发挥大企业集团的作用，加大利用外资力度。

　　（四）加快文化名市建设，推动文化大发展大繁荣。发挥商丘文化底蕴丰厚、文化资源丰富的优势，紧紧围绕“商”字做文章，叫响“华商之都”城市品牌，增强文化产业的软实力和竞争力。

　　大力发展文化事业。加大城乡文化基础设施建设力度，全面加强县级文化馆、图书馆和乡镇（社区）文化站、村级文化大院建设。推进文化信息资源共享、广播电视村村通、农村电影放映和新农村书屋工程，不断满足人民群众多样化的文化需求。加强非物质文化遗产保护。积极推进隋唐大运河商丘段和古城申报世界文化遗产的前期工作。精心组织2008北京奥运会火炬在我市的传递活动，切实增强责任感和使命感，通过参与这一百年盛事，展示商丘形象，推动各项工作。

　　加快发展文化产业。充分发挥“华商之都”城市名片的品牌效应，着力挖掘“商”文化的内涵，研究论证并着手建设一批标志性文化产品，不断提高商丘的知名度、美誉度和影响力。要大力培育特色文化专业村和特色文化产品，弘扬发展民间艺术文化，积极挖掘四平调等民间剧种，剪纸、刺绣等民间工艺，巩固经营性文化事业单位转企改制成果，做大做强演艺集团、报业集团，提高经济效益和社会效益。要放开发展社会文化产业，鼓励引导社会资本进入演艺、鉴赏、旅游和书报音像制品等文化产业领域，推进各类文化企业连锁化经营，不断壮大文化产业规模。

　　着力构建和谐文化。加强社会主义核心价值体系建设，深入开展社会主义荣辱观教育，弘扬民族精神和时代精神，重视社会公德、职业道德、家庭美德和个人品德建设。加强未成年人思想道德建设和大学生思想政治教育工作。开展社会诚信教育，探索建立失信惩戒制度。加强网络文化管理，切实保护知识产权，打击非法出版物，依法取缔黑网吧、黑游戏厅，净化文化市场。深入开展文明行业、文明单位和文明社区创建活动，组织开展科教、文体、法律、卫生进社区和文化、科技、卫生“三下乡”活动，提高文明素养。

　　（五）加快推进城镇化进程，提升城市发展与管理水平。以统筹中心城市、县城、乡镇协调发展为目标，以创建国家园林城市为突破口，加强规划管理，坚持建管并重，完善城市功能，提高城市品位。到2008年底，全市城镇化率达到33．5％。

　　用科学规划引领城市建设。建立健全城乡规划体系，增强规划的科学性和可操作性。严格规划管理，做好《城乡规划法》的宣传和落实工作，确保规划的权威性、约束性和严肃性。合理确定城镇规模、功能定位，体现城镇特色，注重环境容量和节能减排，形成优势互补、各具特色的城镇格局。

　　建立城镇建设推进机制。落实城镇建设主体责任，强化部门协调联系，保证各个部门和环节工作的高质量、高效率。完善建设资金筹措机制，更多地吸引金融机构、大企业和战略投资者参与城镇开发建设。依法规范工程招投标，运用市场机制提高拆迁效率，降低拆迁成本。引导建设单位集中优势力量，改进作业方式，确保如期完成建设任务。

　　完善城市基础设施。建成长江路运河桥和包河桥，新建长江东路等3条市政道路，改造凯旋路中段、民主路西段、文化东路、平原北路，安装和改造长江东路、香君东路等路段路灯。加大道北旧城开发和“城中村”改造力度，加快开发改造步伐。实施火车站广场改造工程，提升城市“窗口”形象。继续抓好运河综合整治工程，优化城市生态环境。优先发展城市公共交通，改善市民出行条件。搞好文化、娱乐等公用设施建设，不断完善城市功能。

　　争创国家园林城市。加快提高城市绿化水平，实施包河、运河市区段两侧绿化和南城湖景观绿化工程。新建8块、改造10块街头公共绿地。对阏伯路、神火大道、南京路、文化路、凯旋路和民主西路等道路进行绿化改造，对商虞大道、归德路南伸段、金桥路等6个城市出入口通道实施绿化。新增公共绿地面积63公顷，人均公共绿地面积由8．16平方米提高到9．12平方米。

　　加强城市精细化管理。增强街道社区自我管理服务功能，探索推行重点区域和部位保洁管理市场化运作机制。加强对城镇居民的教育，提高文明素质。强化城建和市场行政执法队伍建设，提高依法行政、文明执法的能力和水平。继续开展综合整治和专项治理活动，全面清除城市出入口、城乡结合部及背街小巷的“脏乱差”死角，创新管理办法，建立长效机制，提高管理水平。

　　（六）大力发展现代服务业，不断优化经济结构。第三产业的发展水平，是衡量现代社会经济发达程度的重要标志。要围绕我市第三产业整体上还不强不优、规模偏小、层次偏低的问题，着力提高服务业的比重和水平，以消费结构升级带动产业结构调整，增强消费对经济的拉动作用。

　　加强区域性物流中心城市建设。编制好物流园区发展详细规划，启动豫东综合物流园区前期工作。充分发挥全国交通枢纽城市优势，加快发展交通运输业。科学规划和发展各类市场，进一步完善市场体系，促进市场繁荣，加快国家级战略物资储备中心和中储粮商丘直属库、睢阳库、宁陵库异地扩建等项目建设。积极推进郑州至商丘成品油管网建设以及成品油储备库二期工程，提高成品油储备能力。全面推进“万村千乡市场工程”建设，继续支持邮政企业利用网络优势开展连锁配送业务，实施好“家电下乡”工程，努力扩大农村消费。

　　做大做强旅游产业。加强旅游景区基础设施建设和创A工作，搞好芒砀山景区建设和商丘古城、黄河故道生态旅游综合开发，努力打造旅游品牌。加大旅游资源宣传和推介力度，不断扩大旅游景点知名度，增强吸引力。加强旅游行业管理，不断提高旅游接待水平。大力开发旅游商品，拉长旅游产业链条，把旅游业培育成新的经济增长点。

　　加快发展现代服务业。积极发展金融、证券和保险业，深入开展信用体系建设和创建全国金融生态环境模范试点城市活动。加快城信社改制步伐，确保各项指标达到城市商业银行标准，加大支持经济发展的力度。大力发展信息产业和电子商务。发展包装、中介服务、传媒、技术咨询、教育培训、物业管理、家政服务等新型服务业，带动住宿、餐饮、文化娱乐、医疗保健、体育健身等传统服务业提升发展水平。

　　（七）加强生态文明建设，增强可持续发展能力。我市资源型工业比重大，农村环境条件差，在推进工业化、城镇化和农业现代化的进程中，必须围绕节约资源、保护环境、治理污染，走生产发展、生活富裕、生态良好的可持续发展道路。

　　切实抓好节能减排。继续实行严格的节能减排工作责任制和问责制，把节能减排目标落实到各地方、各单位和各企业，跟踪监督，严格检查，严格考核，严格奖惩兑现。严格落实“一票否决”制，强化领导责任和企业法人责任。采取有效措施，加强城乡垃圾收集清运处理，搞好农村面源污染治理。加强建筑领域节能，推广新型墙材。积极开展节约型机关、节约型社区和节约型企业创建活动，建设节约型社会。

　　大力发展循环经济。抓住循环经济试点机遇，着力引进一批循环经济项目，大力推进工业废渣、废气、废水等废弃资源的再生利用。加强污染源控制、水资源保护、废弃物循环利用等工作，促进资源的可持续利用。加快市污水处理厂中水利用工程和生物能源项目建设，加强农作物秸秆等废旧资源的转化增值。

　　推进土地资源集约节约利用。实行严格的土地管理制度，依法管理农村集体和个人建设用地，搞好“空心村”整治，引导农民建房相对集中，少占土地。加强永城采煤塌陷区治理，搞好土地复垦和生态修复，积极创造条件，做好群众搬迁安置工作。坚持工业项目用地的集约发展。深入开展城市违法用地和违法建设集中整治活动，坚决依法制止和查处各类违法用地、违法建设行为，保护土地资源。强化土地收购储备，严格控制土地一级市场，搞活土地二级市场。巩固砖瓦窑场整治成果，市区和县城全面禁止使用实心粘土砖。

　　加快林业生态市建设步伐。突出林业在生态建设中的主体地位，提高造林质量。完成林业生态建设25．91万亩的任务，其中新造林17．65万亩，更新改造4．7万亩，完善提高3．56万亩。进一步完善农田林网，更新改造围村林、环城防护林和工业原料林。加快黄河故道综合开发，把林业生态建设和旅游开发相结合，形成集生态、经济、景观、社会效益于一体的绿色屏障，逐步建成生态旅游开发带。各县（市、区）要以创建林业生态县为载体，优化林业建设布局，加快造林绿化步伐。

　　（八）积极发展社会事业，推进和谐商丘建设。按照党的十七大提出的使全体人民“学有所教、劳有所得、病有所医、老有所养、住有所居”的要求，结合商丘实际，突出重点，扩大覆盖，扎实有效的构建和谐社会。

　　加强就业和社会保障工作。认真落实就业和再就业优惠政策，以培训促进就业，以创业带动就业，统筹城乡就业，重点做好农村富余劳动力的转移就业，帮助零就业家庭解决就业困难，制订优惠政策为全民创业提供支持。规范企业单位用工行为，切实维护劳动者尤其是农民工的合法权益。加大对弱势群体和低收入人群的帮扶力度，切实保障困难群体基本生活。加强农村敬老院建设，农村五保老人集中供养率达到40％。

　　优先发展教育事业。巩固完善基础教育，提高农村义务教育阶段中小学生人均公用经费标准和贫困家庭寄宿生生活费补助标准。从今年秋季学期开始，全面免除城市义务教育阶段学杂费。大力发展职业教育，抓好永城、夏邑、虞城、民权四个劳务输出基地县的技工学校建设，建成国家级农民工培训基地。稳步发展高等教育，着力提高教学质量。积极发展民办教育，鼓励和规范社会力量办学。规范学校收费行为，坚决治理教育乱收费。

　　积极发展医疗卫生事业。加快建立健全覆盖城乡居民的公共卫生服务、医疗服务、医疗保障和药品供应体系，提高医疗服务水平，缓解居民群众看病难、看病贵问题。加大传染病预防控制力度，认真落实结核病、艾滋病等重大传染病防治措施，提高突发公共卫生事件应急处置能力。积极发展社区医疗卫生服务，努力办好惠民医院。

　　全力维护社会稳定。深入开展平安创建活动，加强社会治安综合治理，依法打击违法犯罪。加强人民来信来访工作，积极为困难群众提供法律援助，坚决防止和纠正损害群众合法权益的行为，有效预防和化解各类社会矛盾。严格落实安全生产责任制，做好安全事故隐患的排查和整治工作。加强产品质量和食品药品安全监管，保障人民群众健康安全。建立健全人口与计划生育工作长效机制，提高出生人口素质，稳定低生育水平。重视发展老龄事业，依法保障妇女儿童和未成年人合法权益，关心和支持残疾人事业。发挥工会、共青团、妇联等人民团体桥梁纽带作用，调动社会各方面的积极性、创造性。广泛开展民族团结进步活动，依法管理宗教事务。深入开展国防教育，扎实做好双拥共建工作，大力支持驻商部队和民兵预备役建设，巩固和发展军政军民团结。

（九）切实办好“十件实事”，致力改善民生。从解决社会最关心的难点问题入手，着力办好关系群众切身利益的十件实事。（1）投入2860万元，用于“两免一补”、农村劳动力转移培训、农机具购置等惠农政策补贴，使广大农民群众得到更多实惠。（2）投入1000万元，对城镇居民医疗保险予以补助；投入4100万元，完善新型农村合作医疗制度，使农民参合标准由每人50元提高到100元，建立覆盖城乡的医疗保障体系。（3）投入3000万元，在市区新建4所中小学，缓解市区中小学生上学难问题。（4）投入1100万元，实施农村安全饮水工程，解决25万人的安全饮水问题；开工建设国家面粉及制品质量监督检验中心和市食品药品检验中心，鼓励支持食品药品连锁和集中配送向农村发展，建立农村药品监督网和供应网，保障人民群众饮水、食品和用药安全。（5）投入3000万元用于市区绿化；开工建设睢阳公园和青年公园，人民公园免费向市民开放。（6）做好污染防治工作，开工建设市污水处理厂二期工程和运河上游万吨污水处理厂；筹资7500万元用于林业生态市建设，优化城乡居民生活环境。（7）新改建农村公路1140公里，提高县乡公路通行能力；加强农田水利配套建设，实施“井井通电”工程。（8）改善城市低收入家庭住房条件，全市开工建设经济适用住房32万平方米，享受廉租房补贴的家庭扩大到1．5万户；加快儿童福利院和流浪乞讨人员救助站建设进度。（9）实施农村电影放映工程，全年放映电影4万场次以上，确保全市80％以上的行政村村民每月能看上一场免费电影。（10）开工建设博物馆、科技馆和城市规划展览馆。

四、切实加强政府自身建设

　　为政之道，强在自身。全市各级政府肩负着时代的重托、发展的使命、人民的期待，须臾不可懈怠。必须把依法行政作为为政之要，把改善民生作为为政之本，把真抓实干作为为政之基，把清正廉洁作为为政之德，大力加强政府自身建设，不断提高领导科学发展、和谐发展、跨越发展的能力和水平，奋力开创各项工作新局面。

　　（一）坚持一心为民。人民群众是我们的衣食父母，政府的一切权力都是人民赋予的，执政为民是各级政府的崇高使命和神圣职责，实现好、维护好、发展好最广大人民的根本利益，是政府一切工作的出发点和落脚点。我们一定要把为人民服务视为自己的天职，以争做人民满意的公务员作为自己的追求，多献利民之策，多出利民之举，多办利民之事，让广大群众充分享受改革发展的成果，真切感受党和政府的关怀与温暖。

　　（二）严格依法行政。遵守宪法和法律，是我们政府开展一切工作的根本原则。我们要严格按照法定权限和程序行使权力、履行职责，规范行政执法，完善行政监督，建设法治政府，提高依法行政能力，不断改进政府工作。要认真贯彻落实省委、省政府和市委的决策部署，积极执行人大及其常委会的决议、决定，自觉接受人大的工作监督和法律监督。要主动加强与人民政协的协商，认真听取各民主党派、工商联、无党派人士和人民团体的意见和建议，接受社会公众监督。要带头坚持和完善民主集中制，对涉及群众利益和公共政策的重大事项，广泛听取各方面意见，促进科学决策和民主决策。要健全监管机制，强化责任追究，做到有权必有责、用权受监督、违法要追究。

　　（三）始终务实苦干。求真务实、埋头苦干，是确保政府工作落实的重要保证。做好今年的工作任务艰巨，需要我们静下心来认真干事，多研究解决一些全局性、前瞻性的问题，多做一些打基础、管长远的工作，把全部心思和精力用在谋划大事上，用在推动发展上，切实做到想问题、做决策、办事情，坚持从实际出发，按照客观规律办事，立足当前，着眼长远，努力创造出经得起实践、群众和历史检验的工作成绩。要改进文风、会风和政风，提倡开短会、讲短话、发短文，集中精力抓落实，提高工作质量和工作效率。带头深入一线，靠前指挥，多到条件差、困难多的地方指导工作，帮助解决问题，以实实在在的作风，促进实实在在的发展。

　　（四）切实廉洁从政。始终牢记“两个务必”，保持不骄不躁、艰苦奋斗的作风，严于律己，廉洁奉公，清清白白做人，干干净净做事。要认真落实党风廉政建设责任制，把反腐倡廉建设放在更加突出的位置，旗帜鲜明地反对腐败。推行阳光政务，坚持用制度管人、制权、理事，对经营性土地使用权出让、政府采购、国有资产转让、政府投资项目建设等，要加强管理，规范行为，确保权力依法公开运作和始终受到制约监督。着力构建决策权、执行权、监督权相互制约又相互协调的权力运行机制，从源头上预防腐败，严肃查处以权谋私的违纪违法案件。要加强对政府工作人员的教育、管理和监督。坚持勤俭节约，反对铺张浪费，把有限的资金和资源用在推动经济社会发展上，用在为人民群众谋利益上。

　　各位代表，宏图凝众志，同心谱华章。在新的历史起点上，不断开创跨越发展的宏伟事业，是时代赋予我们的神圣使命，是人民群众的殷切期盼。让我们在省委、省政府和市委的正确领导下，以党的十七大精神为指导，全面贯彻落实科学发展观，团结和依靠全市人民，万众一心、励精图治、求真务实、奋力拼搏，加快建设“经济强市、文化名市、和谐兴市”，共同开创商丘更加美好的明天！
